# Uterocutaneous fistula due to held placenta in a low resource setting

**DOI:** 10.1002/ccr3.1504

**Published:** 2018-04-06

**Authors:** Francesco Di Maggio, Joel Nkurunziza, Paola Caravaggi

**Affiliations:** ^1^ Queens Hospital London UK; ^2^ Department of Surgery Mutoyi Hospital Gitega Burundi

**Keywords:** Held placenta, surgery in austere environment, tropical surgery, uterocutaneous fistula

## Abstract

Postcesarean section complication rate is higher in LMIC (Low and Middle Income Countries) due to lack of resources and specialists availability. A completely or incompletely held infected placenta might underlie a dehiscent cesarean section wound. Humanitarian and local surgeons should consider this differential diagnosis and be ready to practice hysterectomies when needed.

## Introduction

A western surgeon enrolling on a humanitarian mission in a Less Developed Country has to face “lack of resources” to its whole extent 1. This often includes not only high definition scans and mini invasive equipment, but also past medical history data, biochemistry and pathology results.

While dealing with significant communication barriers, only minimal amount of information will be available to support clinical judgment and dictate following therapeutic strategies.

We hereby present an interesting case whose unusual presentation challenged our diagnostic skills.

## Case Report

This 32‐year‐old woman presented to Mutoyi Hospital (Burundi) outpatient clinic complaining of persistent abdominal pain and purulent PV discharge. She had undergone a cesarean section in another hospital 3 months before. To that date, the Pfannenstiel incision had been allegedly resutured twice. Unfortunately, as often in these settings, no medical records of the previous admissions were available.

**Figure 1:**
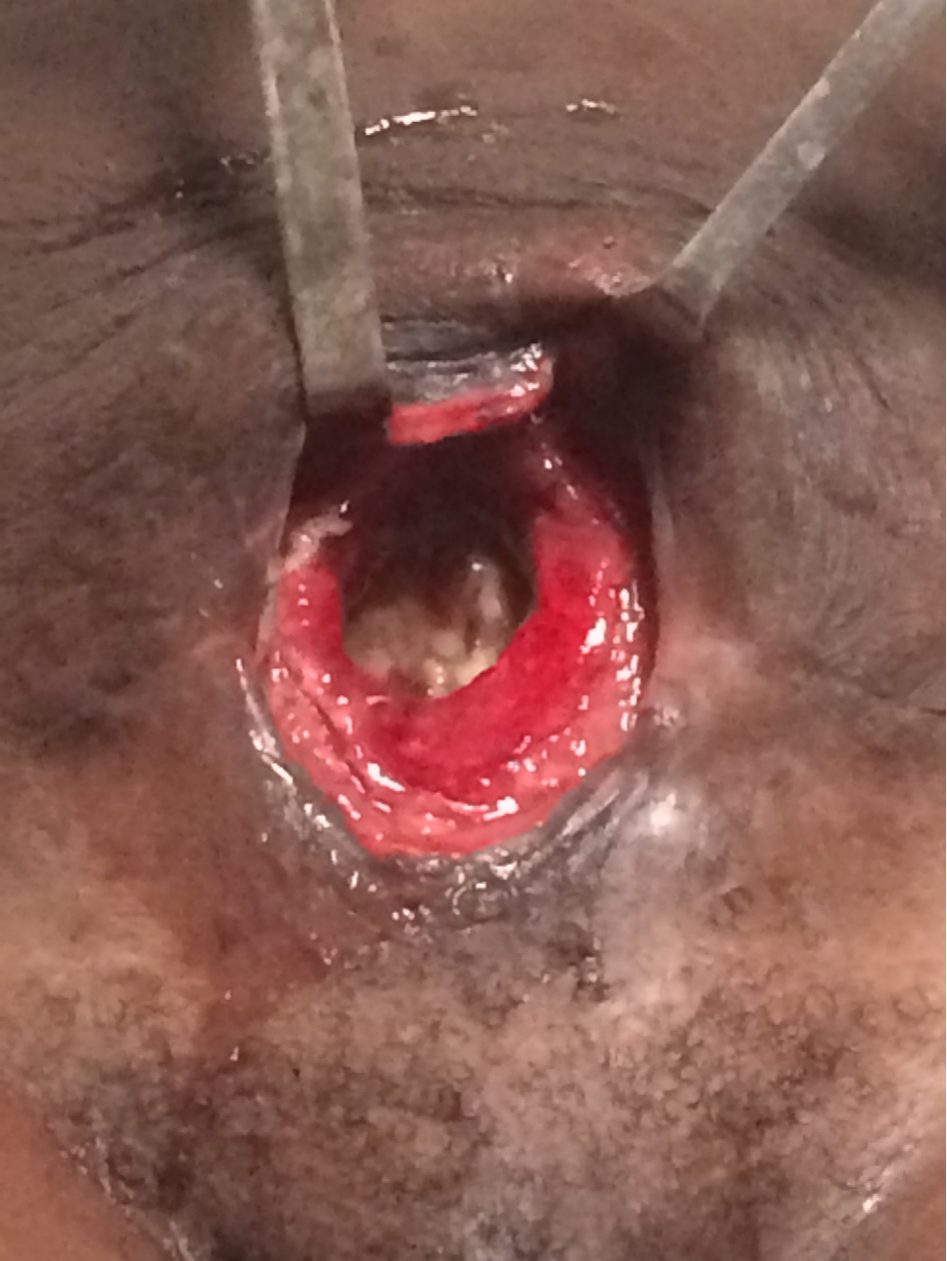
Uterocutaneous fistula.

She presented to our attention afebrile and tachycardic (95), otherwise stable with no rigor. Clinical examination revealed a soft abdomen, tender to palpation in the mesogastric region but without signs of peritonism, and with a 4 × 3 cm open wound in the suprapubic area (Fig. 1).

The blood tests available showed an increased WCC of 14 and Hb of 9 mg/dL, to consider normal in these settings.

The US scan revealed no intra‐abdominal fluid, but some thick tissue (possible a solid viscous) underneath the wound.

**Figure 2:**
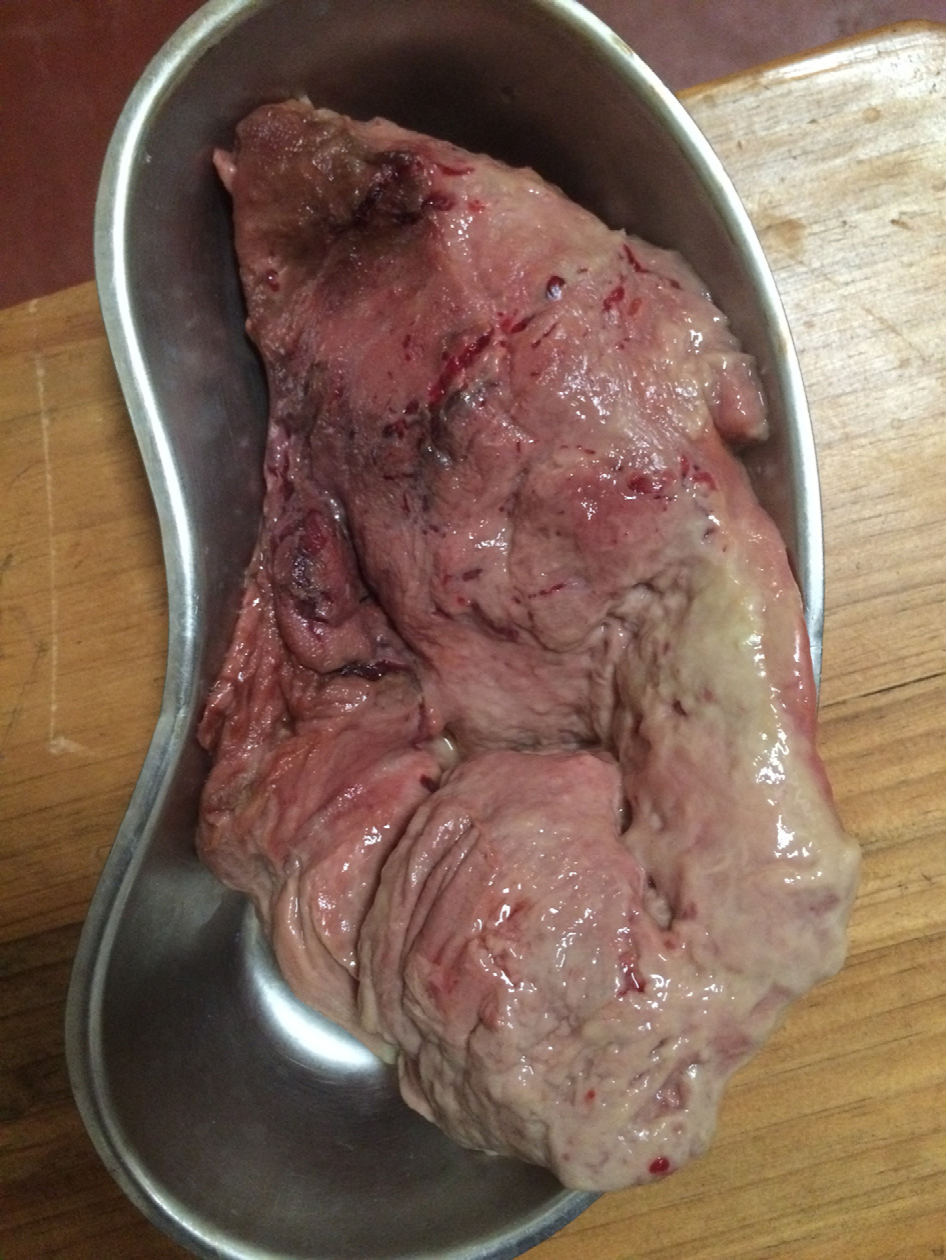
Placenta, as delivered.

On the first day after admission, she delivered from that same wound what we understood could only be a held infected placenta, measuring >15 × 10 × 2 cm (Fig. 2).

As we realized the uterus was communicating directly and largely with the outside, we opted to perform an emergency laparotomy.

The decision was taken to treat the uterocutaneous fistula with a hysterectomy, as the chronically inflamed uterine tissue was unsuitable for repair without tension and would have exposed the patient to a high risk of ruptured uterus in the future.

The patient subsequently underwent a successful emergency hysterectomy and was discharged 10 days later in the absence of complications.

## Authors Comments

Burundi is being consistently rated amongst the three poorest countries in the world. With more recent political issues affecting the country, the latter is possibly an underestimation.

Cesarean sections in that region are often performed in district health centers/ambulatory settings by nurses or local general doctors trained to a very different standard than the one we are used to. The question whether the placenta was intentionally left behind is arguable as the management of a placenta accreta in those settings is unavoidably a hysterectomy 2, 3.

It is nearly impossible to have any sort of clinical records of patients belonging to rural tribes of Burundi. Even in the unlikely case that some discharge document was given to the patient by the previous hospital, it is not part of the local population culture to keep these documents safe/bring them to the hospital in the future.

For the presented case, the information available at the time of admission was collected from a family member who could speak some French and with the help of the local Barundi healthcare providers.

The only blood tests we could perform in Mutoyi included a “normal” Hb of 9 and a mildly raised WCC to 14. The obsolete US machine revealed no fluid but a possible solid organ underneath the wound. There is no MRI scanner available in the whole of Burundi, to the author awareness.

This is very exemplificative situation of what means working in extremely low resource settings.

The case was managed by hysterectomy, because the anterior wall of the uterus was tenaciously adherent to the anterior abdominal wall and in direct communication to the skin via a fistula of 4 cm of diameter (Figure 1). We felt that we could not even attempt to repair the chronically inflamed uterine tissue with a direct suture.

Even in the unlikely event of a successful repair, considering the major challenge represented by birth control/contraception in those settings, the patient would have been exposed to an almost certain new pregnancy with the significant risk of uterine rupture and possible death.

Unfortunately, because there is not a pathology laboratory in Mutoyi hospital, a histological report does not exist.

Lacking important in pieces of information, this manuscript has its strength its authenticity and (hopefully) usefulness to surgeons willing to experience practicing in such a completely different scenario.

## Authorship

FDM: treated the patient in A&E and ideated/wrote the manuscript; JN: treated the patient in A&E and on the ward and assisted the surgery; PC: performed the surgery and treated the patient on the ward.

## Conflict of Interest

The authors declare no conflict of interest.
